# A unique case of acute brain haemorrhage with left ventricular systolic failure requiring ECMO

**DOI:** 10.1186/s12887-019-1658-5

**Published:** 2019-08-13

**Authors:** Kristy Xinghan Fu, Beatrice Hui Zhi Ng, Melissa Hui Xin Chua

**Affiliations:** Khoo Teck Puat - National University Children’s Medical Institute, National University Health System, Singapore, 1E Kent Ridge Road, Singapore, 119228 Singapore

**Keywords:** Takotsubo syndrome, Takotsubo cardiomyopathy, ECMO, Seizures, Acute brain injury, Paediatric

## Abstract

**Background:**

Acute left ventricular (LV) systolic failure as a consequence of acute severe brain injury with status epilepticus in a young infant is not common; managing such a patient on extracorporeal membrane oxygenation (ECMO), which requires proper anticoagulation adds further substrate to a particularly intriguing and novel case worthy of reporting. Takotsubo syndrome and its peculiar clinical presentation is not commonly reported in the paediatric population, yet the high likelihood of this diagnosis joining the dots up for this case invites our curiosity and reflection through the clinical management of this case.

**Case presentation:**

A previously healthy 9-month-old local Chinese boy presented with generalised seizures secondary to acute severe brain injury, with signs of sympathetic overdrive, followed by rapidly progressive cardiogenic shock and respiratory failure, eventually requiring ECMO support. Neuroimaging at presentation revealed bilateral subdural haemorrhages. His cardiac function recovered within the next 24 h revealing the reversibility nature of Takotsubo cardiomyopathy.

**Conclusions:**

This is a captivating case depicting a series of unfortunate and unpredictable clinical events occurring in a previously well infant, which at initial presentation challenged the managing team with regards to its exact aetiology of acute brain injury and acute cardiorespiratory failure. Consideration of various differential diagnoses and finally narrowing down to that of stress-induced reversible cardiomyopathy (Takotsubo syndrome) following his intracranial bleed, versus that of coexisting dual pathology – acute brain injury with concomitant acute viral myocarditis, deepened our understanding of the pathophysiology of each disease process, and how it possibly interlinks between different organ systems.

## Background

Takotsubo syndrome presents as an acute coronary syndrome characterised by severe left ventricular (LV) dysfunction that typically recovers spontaneously within days or weeks.[1, 2] Since Sato et al. originally described it in the 1990s, there have been several case reports and series describing the experience of various centres with this entity with different forms of causative stress – emotional or traumatic physical stress. About 90% of patients described to have Takotsubo syndrome are postmenopausal women. There is limited data of this syndrome in the paediatric population – till date, there are less than 30 reported paediatric cases since Takotsubo syndrome was first described. To our knowledge, the utilisation of extracorporeal membrane oxygenation (ECMO) support in children with possible Takotsubo syndrome has not been reported prior to this. We detail here a case of suspected Takotsubo syndrome resultant from an acute severe brain injury which required ECMO. Through the demonstration of this case and its subsequent progress and management, we aim to illustrate the interdependence of different organ systems and theorise the underlying pathophysiology of this disease process.

## Case presentation

A previously healthy 9-month-old local Chinese boy presented to emergency department with acute onset of generalised seizures. He was well except for mild upper respiratory tract symptoms with intermittent low-grade fever a week prior. There was no reported history of trauma. The seizures had started at his childcare centre after receiving a bath and milk feed. Emergency Medical Services (EMS) was activated and arrived approximately 20 min later. His childcare teacher, who had no prior basic life support training, commenced chest compressions and rescue breathing prior while waiting for EMS, as he appeared off-colour during the seizures. Paramedics on arrival assessed that he had a pulse and cardiac output; therefore he was given rescue breaths via bag-valve-mask ventilation en-route to the nearest emergency department.

He was brought to the nearest general hospital with no specialised paediatric services. His vital signs were: heart rate of 144 beats/minute, blood pressure of 130/72 mmHg, SpO2 was 83% on bag-valve-mask ventilation by paramedics, and axillary temperature was 36 degrees Celsius. As he continued to have more generalised tonic-clonic seizures, he was intubated with rapid-sequence-intubation and mechanically ventilated thereafter. He also received 10 ml/kg of normal saline fluid bolus for persistent tachycardia and poor peripheral perfusion. He was started on midazolam infusion and was transferred to our Children’s Emergency for further management.

**Fig. 1:**
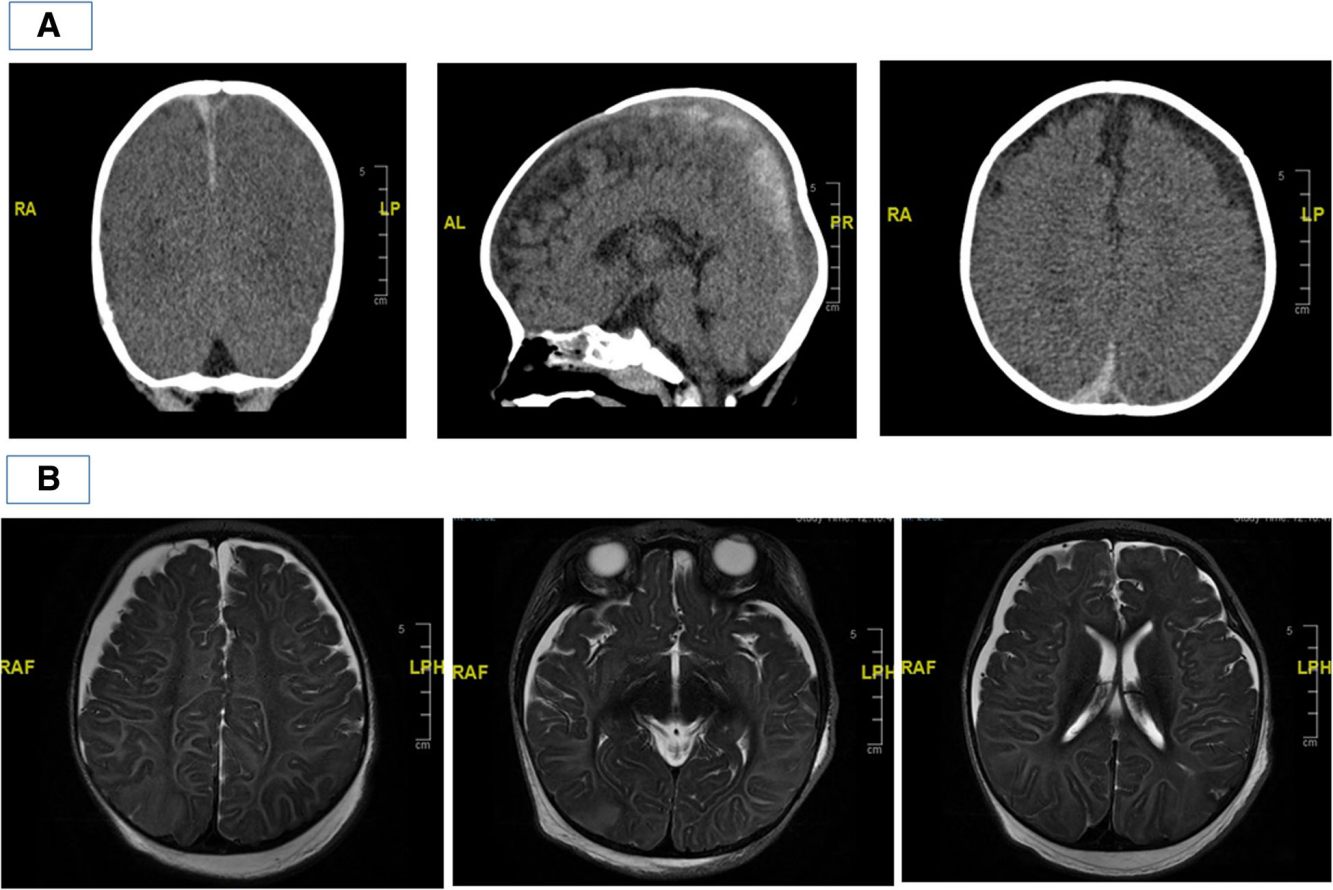
**a** CT brain done at Children’s Emergency showing bilateral subdural haemorrhages. **b** MRI brain done two days off ECMO (T2-weighted images) showing patchy areas of cortical swelling and reduced grey-white differentiation in the respective lobes; leftmost image: right parietal lobe; middle image: occipital lobes; rightmost image: left parietal lobe

At our Children’s Emergency, about 45 min after initial resuscitation, his vital signs were reflective of ongoing sympathetic overdrive. He was severely tachycardic (heart rate was 180 beats/minute) and markedly hypertensive despite repeated non-invasive blood pressure measurements from all limbs (blood pressure was 191/120 mmHg). He also had frequent desaturations to SpO2 80 to 90% (on FiO2 100% via bag-and-mask ventilation) with audible leak. He was poorly perfused but central pulses were present. Bilateral diffuse crackles were heard on lung auscultation, and there was large amount of frothy endotracheal tube (ETT) secretions that required frequent tube suctioning. Dual heart sounds were appreciated with no murmur. Liver edge was felt 1 cm below right subcostal margin, and no organomegaly was noted. Neurological examination revealed generalised hypotonia, areflexia, with no plantar response elicited, likely due to residual muscle relaxant effect. There were no external injuries found. Fundoscopy was not possible as his pupils were pinpoint due to ongoing midazolam infusion. Further history from his childcare teacher and both parents did not suggest an apparent unifying diagnosis for his clinical presentation then. Computed topography (CT) scan of the brain showed acute subdural haemorrhages along the posterior falx, left tentorial leaflet and overlying both high parietofrontal convexities. No significant mass effect, hydrocephalus, brain herniation or acute territorial infarct was noted (Fig. 1a). As he continued to have frequent recurrent desaturations despite ETT suctioning which by then yielded blood-stained frothy secretions, he was re-intubated with Size 4.5 cuff ETT (previous ETT was Size 4, uncuff), as these events were attributed initially to high leak and resultant inadequate ventilation. Prior to transfer to the paediatric intensive care unit (PICU), his blood pressure had returned to normal values, but his SpO2 continued to fluctuate between 80 to 90% on FiO2 100% despite high ventilatory pressures. Neuroprotective measures were instituted.

**Fig. 2:**
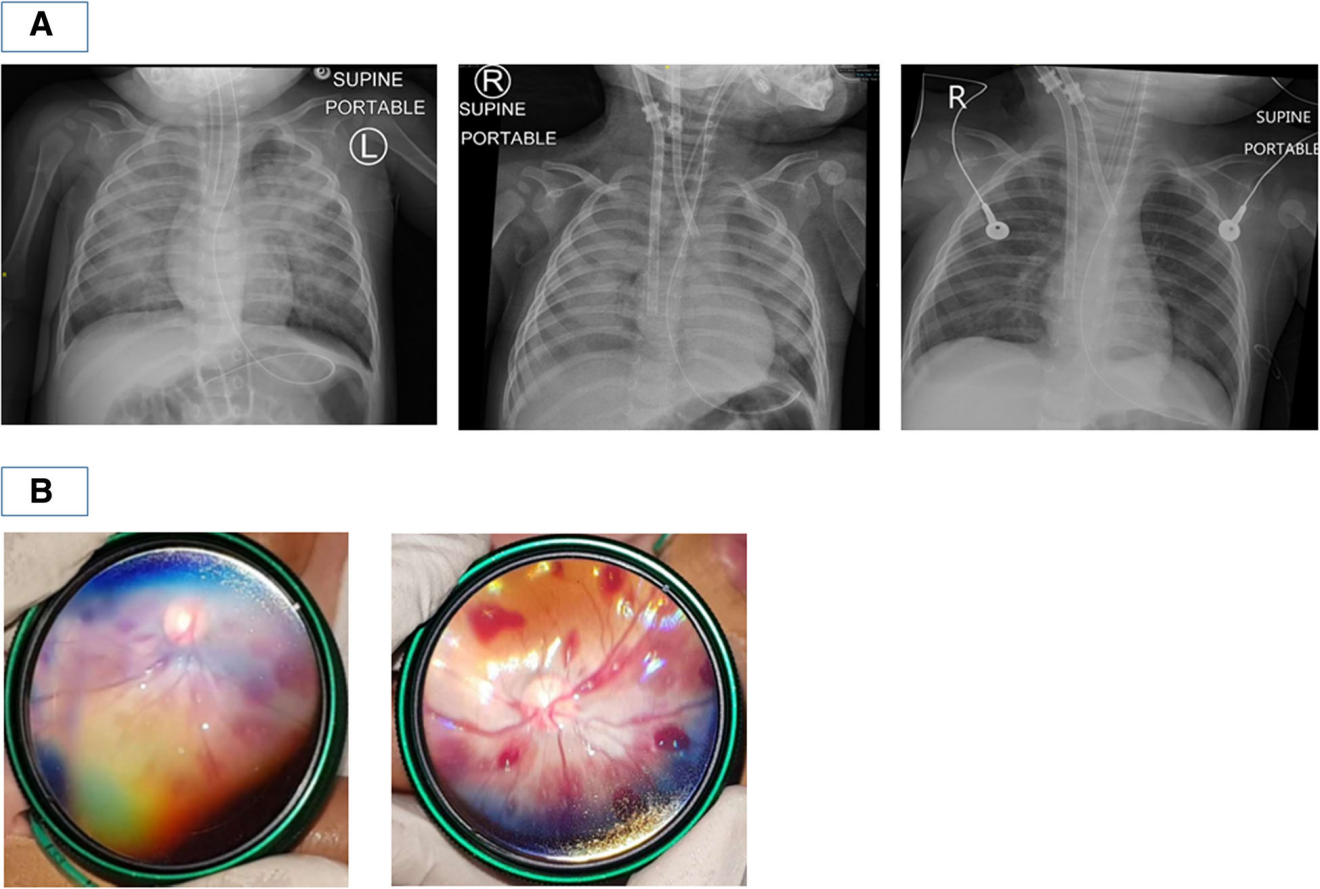
**a** Serial Chest X-rays done showing rapid reversal of pulmonary oedema: leftmost image: done in Children’s Emergency after change of endotracheal tube showing radiographic findings of pulmonary oedema; middle image: done immediately after ECMO cannulation; rightmost image: done the following day, less than 24 h after initiating ECMO. **b** Slit-lamp examination showing extensive acute bilateral intra-retinal haemorrhages; left image: left retina; right image: right retina

On transfer to the PICU, he continued to have persistent desaturation (SpO2 70–80%) despite adequate ventilation of the patient. Chest X-ray showed bilateral pulmonary airspace shadowing and infiltrates suggesting pulmonary oedema (Fig. 2a). Oxygenation index ranged from 22 to 37.8 in the next 6 h. The patient also continued to have brief clinical seizures and was treated with anti-epileptic medication. He also had worsening haemodynamics, necessitating commencement of inotropic support with adrenaline infusion of 0.03 to 0.1mcg/kg/min to maintain adequate blood pressure.

Bedside transthoracic 2D-echocardiogram subsequently showed moderate to severe reduction of LV systolic function and moderately reduced right ventricular systolic function. LV ejection fraction (biplane Simpson) was scored at 31% and fractional shortening was 25%. Hypokinesia involving predominantly the mid LV to LV apex was noted with a qualitatively dilated left atrium and mildly dilated LV. Normal origins of left and right coronary arteries were seen. There was no structural abnormality. Serum creatine kinase (CK) and creatine kinase-MB (CKMB) were normal at 87 U/L and 5.1 μg/L respectively, but troponin I was elevated at 392 ng/L. N-terminal proB-type natriuretic peptide (NT-ProBNP) was normal at 1223 pg/ml. 12-lead electrocardiogram showed sinus tachycardia with no ST elevation or T-wave inversion.

With the 2D-echocardiogram findings and abovementioned initial investigations, we narrowed the differentials to acquired causes: acute viral myocarditis versus Takotsubo cardiomyopathy triggered by a hyperacute stress response after an acute severe brain injury accompanied by status epilepticus.

Intravenous furosemide was given to offload a dilated LV, and milrinone infusion was started at 0.5 to 0.7mcg/kg/min to reduce LV afterload. The patient continued to deteriorate with type 2 respiratory failure due to refractory pulmonary oedema secondary to LV systolic failure despite best medical management. Within 10 h of admission to the PICU, veno-arterial extracorporeal membrane oxygenation (VA-ECMO) support was initiated. ECMO flow ranged from 0.7 to 0.98 LPM (Cardiac index of 2.0 to 2.1 based on his basal surface area). He was peripherally cannulated via right internal jugular vein and right common carotid artery. The extracorporeal circuit was anticoagulated with systemic heparin. There was no haematological complication, even though he had coexisting bilateral subdural haemorrhages on admission. The pulmonary radiological changes on serial Chest X-rays improved remarkably within less than 24 h (Fig. 2a). Repeat 2D-echocardiogram done in less than 24 h from ECMO initiation also showed satisfactory biventricular free wall function with mildly dyskinetic interventricular septum, which was significantly improved when comparing with the admission scan. Investigations were performed to evaluate for possible infective myocarditis and encephalitis, and all of these yielded negative results (blood and urine cultures, and viral PCR studies). He was on ECMO for total of 57 h, and was subsequently successfully decannulated.

This was a dangerously ill 9-month-old boy who was previously well presenting with several clinical problems – firstly, convulsive status epilepticus as a result of acute severe brain injury for which CT imaging revealed bilateral acute subdural haemorrhages despite not having a known history of trauma; secondly, rapidly deteriorating cardiogenic shock with resultant flash pulmonary oedema resulting in respiratory failure despite being on high ventilatory support, finally requiring ECMO support. Possible differential diagnoses considered for his acute brain injury were that of underlying undiagnosed bleeding diathesis, intracranial vascular malformation, aneurysm or neoplasia, cerebral infections, rare metabolic disorders such as glutaric aciduria, and lastly non-accidental injury (NAI). His initial coagulation profile and full blood count were both normal, and had no previous or family history suggestive of an underlying bleeding diathesis. His clinical presentation, initial white blood cell count and inflammatory markers did not suggest bacterial sepsis or meningitis, and later viral studies did not suggest viral encephalitis. Magnetic resonance imaging (MRI) brain that was done 2 days after decannulation from ECMO showed bilateral subdural haematomas and mild sulcal subarachnoid haemorrhage. There was diffuse symmetrical white and grey matter signal abnormality and focal areas of parenchymal swelling. These features were suggestive of diffuse brain injury, which may have been a result of hypoxic-ischaemic injury, post-seizure changes, toxic and metabolic derangements or post-trauma changes (Fig. 1b). Magnetic resonance angiography (MRA) brain scan and transcranial doppler (TCD) of major cerebral vessels were both normal. Comprehensive metabolic workup did not reveal any underlying metabolic disorder. Slit-lamp examination of the eyes, which was done on the second day of admission, revealed extensive acute bilateral intra-retinal haemorrhages (Fig. 2b), raising the possibility of shaken baby syndrome. A full skeletal survey did not reveal any other bone fractures. A thorough investigation by the local justice system did not uncover any proof of NAI at home or at his childcare centre.

## Discussion and conclusions

In summary, this previously well 9-month-old local Chinese boy presented first with convulsive status epilepticus secondary to acute severe brain injury. He developed signs of sympathetic overdrive thereafter, and within the next 2 h from the onset of seizures, his cardiorespiratory system rapidly deteriorated. He had refractory cardiogenic shock, and as a result of LV systolic failure, he developed acute pulmonary oedema resulting in respiratory failure. This eventually led to the necessity for ECMO support, which quickly reversed his shocked state. His cardiac function demonstrated marked improvement within the first 24 h and returned to normal, and he was decannulated soon after.

This case is highly interesting in its presentation and subsequent progress. The initial differential diagnoses considered for his presentation of acute LV failure with acute pulmonary oedema were: severe septic shock with myocardial depression, acute viral myocarditis, underlying undiagnosed congenital structural or coronary anomalies, hereditary cardiomyopathy, and lastly Takotsubo cardiomyopathy. Following the course of his progress and the investigations done during his hospitalisation, the likelihood of Takotsubo cardiomyopathy was topmost of our differential list, given the quick reversible nature of his LV failure, and the context in which the child had a preceding acute brain haemorrhage. Within the managing teams, there were two schools of thought with regards to concluding the final diagnosis of this patient’s clinical presentation – firstly, postulation that the patient had dual severe concurrent pathology occurring in the same patient at the same time: both an acute severe brain injury resulting in refractory seizures, and acute viral myocarditis resulting in cardiopulmonary failure; secondly, that of stress-induced Takotsubo cardiomyopathy resultant from an acute brain injury complicated by status epilepticus. The clinical entity of stress-induced Takotsubo cardiomyopathy syndrome was more likely in view of the sequential pattern of clinical problems that developed over the first 12 h of clinical presentation and subsequently, the speed of recovery of the patient’s cardiorespiratory system after ECMO initiation, which is typically longer for acute viral myocarditis.

Amongst the cases of Takotsubo cardiomyopathy reported for the paediatric population, there have been several which described a similar reversible stress-induced cardiomyopathy occurring after spontaneous intracranial haemorrhages.[3] What is truly compelling and novel about this case, is that this patient had a unique clinical presentation that demonstrated a sequential pattern of clinical causation which outlined the pathophysiological postulation describing Takotsubo syndrome. Our centre’s experience in commencing the patient on ECMO when faced with refractory cardiorespiratory failure is yet another area that adds to current medical literature; this is, in our knowledge, the first report of ECMO being utilised in a paediatric patient with stress-induced cardiomyopathy from an acute brain haemorrhage. ECMO may not be readily available in all centres, and some clinicians may feel that it would be relatively contraindicated in patients with acute intracranial haemorrhage due to challenges in effective anticoagulation.

Cheah et al. previously described that the brain injury pattern in traumatic brain injury patients presenting with Takotsubo cardiomyopathy is heterogeneous, even though this is more common in patients with subarachnoid haemorrhages.[4] In the majority of patients, inotropic support using dobutamine leads to improved cardiac function. Patients with severe refractory cardiovascular shock may necessitate ECMO support; long-term prognosis is however more dependent on the severity of brain injury.[5] This resembles the clinical course of our patient – even though ECMO was commenced very early, and he was successfully decannulated off ECMO after a relatively short course of support, he went on and developed mixed spastic dystonic quadriplegic cerebral palsy and epilepsy.

Pelliccia et al.^1^ and Wittstein et al.^4^ clearly summarises the current postulated pathophysiology behind Takotsubo syndrome: as acute stressors induce brain activation, there is increasing bioavailability of cortisol and catecholamines, hence resulting in both circulating epinephrine and norepinephrine to be released into the bloodstream. Through multiple mechanisms, this catecholamine surge leads to direct catecholamine toxicity, causing adrenoceptor-mediated damage, epicardial and microvascular coronary vasoconstriction and/or spasm, and increased cardiac workload and finally to myocardial damage eventually. This sequential description is distinctively represented in our patient’s clinical presentation of sympathetic overdrive preceded by an initial brain injury followed by the first hour of resuscitation (acute physical stress), with notable documented persistent tachycardia and hypertension, accompanied with signs of severe vasoconstriction (the catecholamine surge seen in the acute phase of Takotsubo syndrome), and finally the rapidly failing LV systolic function resulting in progressively worsening pulmonary oedema and cardiorespiratory failure.

This case exemplifies the importance of genuinely understanding the pathophysiology of each disease process when considering differential diagnoses for a complex clinical presentation. It provided a deeper understanding of the pathophysiology of Takotsubo syndrome, neurohumoral features in myocardial stunning, and the relationship between acute brain injury and reversible LV systolic failure. Reflecting through the differential diagnoses considered for the clinical problems presented at different time-points of this patient’s medical journey was highly valuable and invigorating for the managing clinicians. This case also opposes the classical teaching that “common things occur commonly”, and motivates us to always think outside the box when faced with an atypical case presentation and a diagnostic dilemma. It may always remain as an intellectual debate regarding the exact aetiology for our patient’s presentation, why he had suffered an acute severe brain injury, and coincidentally (or consequently) developed severe LV systolic failure, which was highly reversible. The lesson learnt to actively consider a wide range of differential diagnoses and to understand pathological process of each disease, is perhaps more valuable than winning the debate eventually.

## Data Availability

All relevant data for the case presented are included in the manuscript.

## Abbreviations

%: Percentage
2D-echocardiogram: 2 dimensional-echocardiogram
CK: Creatine kinase
CKMB: Creatine kinase-MB
CT: Computed tomography
ECMO: Extracorporeal membrane oxygenation
EMS: Emergency medical service
ETT: Endotracheal tube
FiO2: Fractional inspired oxygen
LPM: Litres per minute
LV: Left ventricular
MRA: Magnetic resonance angiography
MRI: Magnetic resonance imaging
NAI: Non-accidental injury
NT-ProBNP: N-terminal proB-type natriuretic peptide
PEEP: Positive end-expiratory pressure
PICU: Paediatric intensive care unit
PIP: Peak inspiratory pressure
SpO2: Saturations by pulse oximetry
TCD: Transcranial doppler
VA-ECMO: Veno-arterial extracorporeal membrane oxygenation
